# The Potential for Foreign Body Reaction of Implanted Poly-L-Lactic Acid: A Systematic Review

**DOI:** 10.3390/polym16060817

**Published:** 2024-03-14

**Authors:** Melanie Nonhoff, Jan Puetzler, Julian Hasselmann, Manfred Fobker, Georg Gosheger, Martin Schulze

**Affiliations:** 1 Department of General Orthopedics and Tumor Orthopedics, Muenster University Hospital, 48149 Münster, Germany; 2 Materials Engineering Laboratory, Department of Mechanical Engineering, University of Applied Sciences Muenster, 48565 Steinfurt, Germany; 3 Central Laboratory, Muenster University Hospital, Albert-Schweitzer-Campus 1, 48149 Münster, Germany

**Keywords:** foreign body reaction, poly-L-lactic acid, implant coating, degradation

## Abstract

Poly-L-lactic acid (PLLA) implants have been used for bone fixation for decades. However, upon insertion, they can cause a foreign body reaction (FBR) that may lead to complications. On 15 December 2023, a systematic review was conducted to search for articles on the PubMed, MeSH term, and Scopus databases using the keywords ‘PLLA’ and ‘foreign body reaction’. The articles were reviewed not only for the question of FBR, its severity, and the manifestation of symptoms but also for the type of implant and its location in the body, the species, and the number of individuals included. A total of 71 original articles were identified. Of these, two-thirds reported on in vivo trials, and one-third reported on clinical applications. The overall majority of the reactions were mild in more than half of the investigations. Symptoms of extreme and extensive FBR mainly include osteolysis, ganglion cysts, and swelling. The localization of PLLA implants in bone can often result in osteolysis due to local acidosis. This issue can be mitigated by adding hydroxyapatite. There should be no strong FBR when PLLA is fragmented to 0.5–4 µm by extracorporeal shock wave.

## 1. Introduction

Poly-L-lactic acid (PLLA) is a biopolymer that has been used for decades in various surgical implants, including screws, plates, and anchors for bone fixation [1,2]. Additionally, it is commonly used as an injectable dermal filler due to its ability to stimulate collagen production [3].

PLLA, a member of the polylactic acid (PLA) enantiomer family, is both biodegradable and biocompatible. Its degradation occurs in two phases: first, the ester bonds are hydrolyzed; second, the product is degraded to lactate, carbon dioxide, and water [4,5]. The rate of degradation is slower than that of other polymers, and it is estimated that absorption takes more than three years [5,6]. The duration is influenced by the polymer’s crystallization, molecular weight, and morphology of the polymer [7]. Compared with its enantiomer poly-D-lactic acid (PDLA), PLLA exhibits a higher degree of crystallinity, although both are semicrystalline. This results in a slower degradation rate in PLLA, which is ideal for maintaining the mechanical stability of implants [8]. The molecular weight of PLLA is inversely proportional to its degree of crystallinity. This results in a proportionality between molecular weight and degradation rate, unlike other PLAs [9].

**Figure 1:**
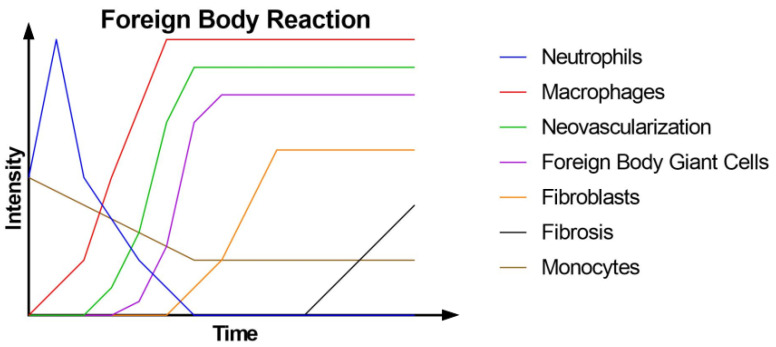
Intensity of different components of the foreign body reaction over time.

The first stage of PLLA biodegradation is linked to a subclinical foreign body response (FBR). Following implantation, the body absorbs proteins, which triggers the FBR. Immune cells, primarily neutrophils at first and then monocytes, infiltrate the tissues surrounding the polymer, initiating an inflammatory response that upregulates cytokines. The monocytes differentiate into macrophages, which attempt to phagocytose the foreign body but ultimately fail. They then fuse together to form multinucleated foreign body giant cells (FBGCs). This process also induces neovascularization and activates fibroblasts to synthesize collagen, which eventually encapsulates the polymer, including the giant cells, leading to fibrosis (Figure 1). During the second phase, the waning effect begins, leading to a gradual decrease in inflammation and the number of immune cells [4,5,10,11]. The capsule is primarily composed of collagen types I and III, and its thickness is positively correlated with the surface/volume ratio of the polymer [10,12,13]. The FBGCs contribute to the degradation of the polymer and are present throughout the implant’s lifespan [13].

Severe FBR may lead to post-implantation complications. However, some degree of FBR is desirable in dermal fillers to achieve the desired volumizing effect and in bone fixation to activate cell differentiation and promote healing through transient inflammation [10,14].

During the development and testing of an anti-infective implant coating based on PLLA mixed with adjuvants [15,16,17], concerns were raised regarding the risk of an FBR triggered by PLLA. This systematic review aims to assess the risk of FBRs caused by PLLA by providing an overview of relevant studies. The severity and circumstances surrounding the reactions are also analyzed.

## 2. Materials and Methods

On 15 December 2023, a search was conducted on PubMed for the terms ‘PLLA’ and ‘foreign body reaction’ in titles and abstracts, which returned 44 results. On the same day, a search was performed on Scopus for the same terms in article titles, abstracts, and keywords, resulting in 109 documents. Additionally, a MeSH Term search was conducted on PubMed for ((“poly(lactide)” (Supplementary Concept)) AND “Foreign-Body Reaction”(Mesh)), which delivered 139 results.

**Figure 2:**
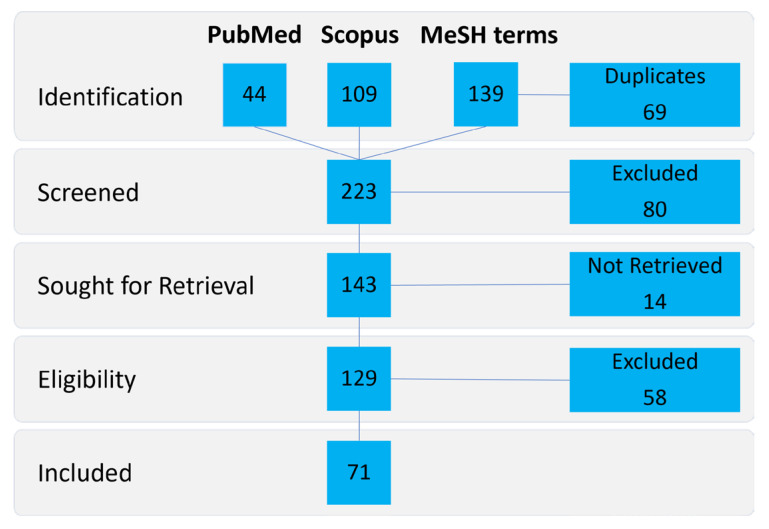
Preferred reporting items for systematic reviews and meta-analyses (PRISMA) flow diagram.

Following the Preferred Reporting Items for Systematic Reviews and Meta-Analyses (PRISMA) guidelines, 69 duplicates were excluded after identification. An additional 80 articles were excluded during screening. These articles included case reports, reviews, book chapters, short conference abstracts, and in vitro studies. Fourteen more articles were unavailable. Of the remaining 129 articles, 58 were excluded because they were in a foreign language other than English or German, pertained to the wrong or unspecific material, or were studies on adverse effects. Therefore, this review comprises 71 articles (Figure 2).

The remaining articles were reviewed to determine the type of study, organism, location within the organism, type and size of implant, particle size, duration of implantation, and number of subjects. The studies were also examined for the occurrence of FBRs, their symptoms and severity, and treatment after an FBR. If there was no mention of any FBR symptoms or a clear statement that it did not occur, the reaction was classified as absent. Extreme or extensive severity indicates the acknowledgment of clinical symptoms of FBR. Extreme severity includes long-lasting and damaging symptoms, such as osteolysis, while extensive severity includes uncomfortable symptoms for the subject such as swelling and pain. The term ‘mild severity’ is used in cases in which the authors labeled the condition as ‘mild’, ‘nonclinical’, ‘noninflammatory’, or similar, or when there were no symptoms other than histologic findings.

## 3. Results

There were 71 unique studies that met the criteria. The publications spanned from 1988 to 2023. Forty-seven studies used in vivo methods. Of these, three used a dog model [18,19,20], three used a goat model [21,22,23], four used a mouse model [24,25,26,27], one used a pig model [28], 17 used a rabbit model [7,29,30,31,32,33,34,35,36,37,38,39,40,41,42,43,44], 12 used a rat model [6,45,46,47,48,49,50,51,52,53,54,55], and 7 used a sheep model [56,57,58,59,60,61,62]. The remaining twenty-four articles were clinical studies. Eight studies were stated as prospective [63,64,65,66,67,68,69,70] and another eight were stated as retrospective [71,72,73,74,75,76,77,78], while the design of the other eight was not specified [79,80,81,82,83,84,85,86].

FBR was completely absent in 20 out of the 72 studies (28.17%; Table 1).

Five of the studies (7.04%) presented extreme cases of FBR (Table 2). Sinisaari et al. (1995) presented 420 applications of various PLLA implants in which one patient developed a non-bacterial sinus formation due to an FBR, resulting in an incidence of 0.24%. This incidence is comparatively low when compared with the other materials used for implants in this study, namely polyglycolide (PGA) and a PLLA/PGA compound (PGA: 3.3%; PLLA/PGA: 1.26%) [81].

Three cases of osteolyses were reported. In Tams et al. (1996), one out of four patients (25%) experienced swelling and osteolysis 5.5 years after an osteosynthesis with a PLLA plate in a mandibular osteotomy. The osteolytic areas disappeared after the removal of the PLLA remnants [82]. Böstman et al. (1998) investigated 234 patients with ankle fractures treated with PLLA screws and pins. Out of these, 85 patients were treated with PGA screws and pins simultaneously. One patient (0.43%) who received both materials returned for ankle arthrodesis at 12 weeks with osteolytic changes and cystic extensions. The osteolysis remodeled within three years. However, the arthrodesis worsened. It is unclear whether osteolysis was more prevalent around the PGA or PLLA. In cases with only PGA or PLLA/PGA compound implants, the incidence rates of similar reactions were 0.85% and 2.08%, respectively [76]. Potapov et al. (2011) conducted a retrospective analysis of 22 cases involving the use of PLLA screws for distal biceps tenodesis. One patient (4.55%) was diagnosed with FBR with osteolysis after developing a painful sinus with swelling at 6 weeks. The screw was removed, resulting in the subsiding of the patient’s FBR [75].

Pistner et al. (1993) conducted an in vivo study in a rat model, which resulted in 3 out of 67 rats (4.48%) developing spindle cell sarcomas. All rats had rods or cubes of PLLA implanted subcutaneously in the back muscle. Mild histologic signs of FBR were observed in all rats, including immune cells, FBGCs, and fibrous capsules [55].

The occurrence of extreme cases of FBR ranged from 0.24% to 25%, decreasing as the number of cases increased.

Eight out of the total number of studies (11.27%) presented extensive cases (Table 3). In a retrospective clinical study conducted by Kim et al. (2019), 3 out of 15 patients (20%) exhibited signs of redness and tenderness two years after internal fixation of zygomaticomaxillary fractures with PLLA-hydroxyapatite (HA) plates. Two patients recovered after receiving non-steroidal anti-inflammatory drugs (NSAIDs), and one patient recovered after the removal of the implant [73].

Swelling was reported as a symptom in four different articles. Bergsma et al. (1993) described the treatment of patients with zygomatic fractures using PLLA plates and screws. Nine out of ten patients (90%) exhibited intermittent local swelling without inflammation. FBR was confirmed through light microscopic analysis. The excess tissue was removed, resulting in a reduction in the reaction [79]. Hayashi et al. (2013) treated 17 patients with frontal bone or zygomatic bone fractures using PLLA-HA plates and screws. Two of the patients (11.76%) exhibited inflammatory granulation tissue, while one of them also experienced swelling after two years. The swelling subsided after the removal of the tissue and the implant [80]. In a prospective study by Landes et al. (2014), PLLA-HA plates were used for various facial fracture fixations in 29 patients. At 15 and 33 months, two patients (6.90%) returned with swelling, which was treated with debridement [63]. In their 2014 retrospective study, Ramsingh et al. examined 268 patients who underwent anterior cruciate ligament (ACL) reconstruction. Screws composed of PLLA and beta-tricalcium phosphate (TCP) were utilized. Fourteen patients (5.22%) reported swelling at the pre-tibial screw site between 12 and 24 weeks after surgery. All patients also exhibited bone marrow edema and ganglion cysts upon further examination [78].

Two additional studies also reported cases of edema and cysts. Lee et al. (2017) compared patients undergoing ACL reconstructions with PLLA or PLLA-HA screws in a retrospective study. With 86 patients in each group, the PLLA group showed 66 edemas and 11 cysts (89.53%), while the PLLA-HA group showed 21 edemas and 2 cysts (26.74%). The authors concluded that tibial tunnel widening caused these reactions and that the additive successfully reduced their incidence [74]. In the retrospective study by Veizi et al. (2022), out of 27 patients with PLLA-HA screws used in ACL reconstruction, 9 patients developed edema and 2 patients developed cysts (40.74%) [71].

In a study on PLLA fillers conducted by Janjua et al. (2014) [70], 91 participants were instructed to either massage the injection site or refrain from doing so. In the group that massaged, 27 participants developed nodules, and in the group that did not massage, 8 participants developed nodules (38.46%).

The incidence of extensive FBR ranged from 5.22% to 90%. The highest number of participants had an incidence of 5.22%, while the lowest had an incidence of 90%.

The majority, namely 39 studies (54.93%), mentioned only mild FBR (Table 4). The histological symptoms consisted primarily of immune cells, such as macrophages and lymphocytes, as well as fibrous tissue or capsules. Additionally, FBGCs were present in 26 of the studies.

No other allergic reactions were mentioned in any of the articles.

### 3.1. Location as a Factor

When examining foreign body reactions caused by implants, it is crucial to distinguish between the reactions of the bone and those of the soft tissue. Of the selected articles, 29 focused on bone reactions (40.84%), 36 focused on soft tissue reactions (50.70%), and 6 examined both (8.45%). FBR was not present in 15 of the articles related to bones (42.86%), and 5 of the articles related to soft tissue (11.90%).

There were four extreme reactions observed in relation to bone defects, which accounted for 11.43% of articles. Osteolysis was observed in three instances (8.57%): during a mandibular osteotomy [82], a biceps tendon repair [75], and an ankle fracture repair [76]. In one in vivo study, rats developed spindle cell sarcomas due to the Oppenheimer effect (2.86%) [55], which will be discussed later. The soft tissue exhibited two extreme cases (4.76%), both sinus formations in the aforementioned biceps tendon repair [75], and another unidentified case [81]. In the extreme cases, all other symptoms were related to the soft tissue, such as swelling, tenderness, and histologic findings.

Three articles reported extensive cases of bone involvement (8.57%), all of which were related to ACL reconstructions and resulted in bone marrow edema and ganglion cysts [71,74,78]. Soft tissue reactions were reported in six articles (14.29%), with four showing swelling (9.52%), one showing redness and tenderness (2.38%), and one showing nodules (2.38%). Swelling was mainly associated with the use of plates in the facial region [63,79,80] but was also noted in one ACL reconstruction [78]. Redness and tenderness were also caused by a facial plate [73]. Nodules were associated with the use of fillers in various regions [70].

Mild cases of FBR were present in bone 13 times (37.14%) and in soft tissue 25 times (59.52%). Parker et al. (2002) compared mild cases in bone and soft tissue and found that subperiosteal implants developed a higher quality of fibrous capsules that reduced in thickness over time, while subcutaneous capsules became thicker. There was no discernible difference in the inflammatory reaction. The authors attributed the variation in capsules to a difference in friction between the implant and the tissue [23].

Generally, most adverse effects occurred in relation to implants inserted near joints and in the face.

### 3.2. Implant and Particle Parameters as a Factor

The only article that mentioned the size of the residual particles that triggered the reaction was an in vivo study by Bergsma et al. (1995) [45]. They compared PLLA, cross-linked PLLA, and PLA96 (a form of polylactide containing 96% PLLA and 4% D-enantiomer), all predegraded, in a rat model degradation study. After 80 weeks, the 5 mm^2^ PLLA capsules (molecular weight: 16.6 kDa; heat of fusion: 82.9 J/g) degraded into needle-like fragments with an average size of 2.742 mm^2^. The FBR was mild for all materials, but PLA96 (molecular weight: 2 kDa; heat of fusion: 60.5 J/g) produced the strongest FBR due to its faster degradation rate resulting from the lower material crystallinity. The average particle size of the pure PLLA was larger than that of the sample under investigation. Cross-linked PLLA also degraded at a faster rate.

#### 3.2.1. Molecular Weight

Out of the 71 articles, 39 reported the molecular weight of the implant used, which ranged from 16.6 to 1650 kDa. There was no reaction in eight articles (20.51%) with a range of 50 to 770 kDa. Two articles (5.13%) were classified as extreme reactions (120 to 950 kDa), one (2.56%) as extensive (1000 kDa), and 28 (71.80%) as mild (16.6 to 1650 kDa).

Three articles compared polymers with different molecular weights. Kunz et al. (1995) found no in vivo differences between 70 kDa PLLA and 205 kDa PLLA, and no reactions were observed [43]. In Pistner et al. (1993), three different molecular weights resulted in mainly mild FBR. The injection-molded PLLA with 120 kDa (inherent viscosity: 1.8 dL/g; amorphous) showed the mildest reaction compared with an injection-molded PLLA with 203 kDa (inherent viscosity: 2.8 dL/g; amorphous) and a PLLA machined from a solid block with 429 kDa (inherent viscosity: 4.9 dL/g; 73% crystallinity). This is the aforementioned study in which three rats developed sarcomas, two of which were implanted with the highly crystalline PLLA and one with the lowest-molecular-weight PLLA. However, Oppenheimer sarcomas can form regardless of the chemical nature of a material, as will be discussed later [55].

Zhang et al. (2021) observed three different molecular weights of PLLA fillers in their research. A subcutaneous injection of PLLA fillers was administered to the backs of rabbits. The fillers had molecular weights of 152 kDa (microsphere size: 20–85 µm; intrinsic viscosity: 1.39 dL/g), 604 kDa (microsphere size: 20–90 µm; intrinsic viscosity: 3.80 dL/g), and 1650 kDa (microsphere size: 5–100 µm; intrinsic viscosity: 6.89 dL/g). After 4 months, the PLLA filler with the lowest molecular weight exhibited the least formation of type I collagen and was already degraded. The PLLA with intermediate molecular weight was described with moderate degradation (9 months) and showed a weak FBR, but it was stronger than that of the PLLA with a low weight. Additionally, this filler resulted in the highest formation of type I collagen. The filler with the highest molecular weight exhibited the longest degradation time and the weakest overall FBR. The level of neocollagenesis observed was exceptionally low. The authors concluded that there is a correlation between the fast degradation of PLLA and a strong, early FBR or slow degradation and a weak, late FBR [7].

Six different studies reported the molecular weight after degradation in the body. Weir et al. (2004) conducted a study on subcutaneous rods in rats. The weight of the rods decreased from 399 kDa to 69.8 kDa after 44 weeks without FBR [54]. Morizane et al. (2019) conducted a study on rabbit knees and found that rods with a molecular weight reduction from 250 kDa to 50 kDa did not produce an FBR after 25 weeks [37]. According to Tams et al. (1996), a facial plate used in a clinical setting was reduced from 950 kDa to 0.85 kDa (highly crystalline; heat of fusion: 89 J/kg) over a period of 5.5 years. The patient experienced an extreme FBR with osteolysis, leading to the removal of the single measured plate [82]. Schakenraad et al. (1988) found that PLLA fibers decreased from 65 kDa to 39 kDa subcutaneously in rats over a period of 6 months. The study observed only a mild reaction with the formation of a fibrous capsule and infiltration of macrophages [48]. Matsusue et al. (1992) noted that rods in the knees of rabbits were reduced from 220 kDa to 1 kDa over a period of 1.5 years. The reaction was mild, with only one FBGC detected and the formation of fibrous capsules [30]. In a biodegradation study conducted by Suuronen et al. (1998), facial plates in sheep were reduced from 49.8 kDa to 3.5 kDa over a period of 5 years. The intrinsic viscosity decreased from 1.46 dL/g to 0.21 dL/g, while the heat of fusion increased from 50 J/kg to 70.5 J/kg and the crystallinity increased from 53% to 75%. The sheep exhibited a mild reaction with FBGCs and mononuclear inflammatory cells [60].

#### 3.2.2. Viscosity

The articles frequently calculated the molecular weight *M_v_* by measuring the intrinsic viscosity [*η*] and applying the Mark–Houwink equation for PLLA [87]:

(1) $$\left[\eta \right]=5.45\times 10^{-4}\times M_{v}^{0.73}$$

It is important to note that the specific polymer parameters for calculation in the formula may vary between the articles. As per the formula, a decrease in the molecular weight of the polymer leads to a corresponding decrease in its intrinsic viscosity and subsequently a slower degradation. Only Zhang et al. (2021) and Suuronen et al. (1998) mentioned their measured intrinsic viscosity values, which were already reported in Section 3.2.1 [7,60].

Four of the articles provided the intrinsic viscosity of the polymer based on the manufacturer’s data. The inherent viscosity is calculated as the natural logarithm of the concentration and the relative viscosity, which is determined by comparing it to the solvents. The inherent viscosities of the study by Pistner et al. (1998) were mentioned in Section 3.2.1 [55]. Sena et al. (2012) used screws with an inherent viscosity of 5.8 to 7.2 dL/g (molecular weight: 1500 kDa) for flat foot surgeries. Out of 33 patients, 13 exhibited mild FBRs with macrophage infiltration, FBGCs, and dense collagenous tissue [84]. Van Dijk et al. (2005) compared two PLLA cages in the vertebrae of goats. The inherent viscosities of the cages were 2.64 and 2.45 dL/g, respectively. The reaction was similar in both cages, with a mild intensity. However, the fibrous capsule was thinner when using 2.64 dL/g-PLLA at 3 and 6 months [21]. Marascalco et al. (2009) conducted subcutaneous trials on rats using scaffolds with an inherent viscosity of 3.2 to 4.3 dL/g. The FBR was mild, with the presence of FBGCs and neocollagenesis [47].

#### 3.2.3. Crystallinity

The percentage of crystallinity, which is inversely proportional to the molecular weight in thermoplastics, was reported in seven of the articles. The values from the studies by Pistner et al. (1998) and Suuronen et al. (1998) are provided in Section 3.2.1 [55,60].

The same material was used in four studies for plates and pins. The molecular weight of the PLLA was 50 kDa, with a 50% crystallization rate. Koskikare et al. (1996) and Pihlajamäki et al. (2010) did not observe a reaction, whereas Peltoniemi et al. (1998) and Peltoniemi et al. (1998) reported a mild reaction characterized by the presence of FBGCs, fibrous capsules, and macrophage infiltration [34,40,58,59].

Puumanen et al. (2001) conducted a study using pins with 65 to 78% crystallinity (molecular weight: 45 to 65 kDa) subcutaneously in rabbits. The FBR was mild, with only macrophage infiltration [41].

In three studies, the crystallinity was expressed as the heat of fusion, which is the enthalpy released during the melting of the polymer. This parameter provides indirect information about the polymer’s crystallinity. The heat of fusion values from Bergsma et al. (1995), Tams et al. (1996), and Suuronen et al. (1998) were previously mentioned [45,60,82].

### 3.3. Duration of Implantation as a Factor

The articles reported varying durations of implantation, ranging from a few days to multiple years. Absent reactions were observed in articles with durations between 38 days and 6 years, while extreme reactions occurred between 1 year and 5.5 years. The range of extensive reactions spanned from 1 year to 6 years and 2 months. Mild reactions, on the other hand, exhibited the greatest variability, ranging from 7 days to 8 years and 8 months. An article that showed no reaction did not mention the duration of implantation [77].

It is difficult to determine the exact course of foreign body reactions due to varying degradation speeds. However, Laitinen et al. (1993) reported a waning or healing effect after 6 weeks, Matsusue et al. (1992) after 78 weeks, Neto et al. (2021) after 3 days, and van Dijk et al. (2005) after 36 months [21,30,51,61]. Generally, all articles stated that after the removal of the remnants or complete degradation, all effects of FBRs disappeared.

### 3.4. Additives as a Factor

Seventeen studies investigated the incorporation of additives into PLLA to attenuate the immune response.

The use of hydroxyapatite, a mineral found in bone, was investigated for its osteoconductivity in eleven studies. Five of the studies reported a complete absence of foreign body reactions (45.45%). Two of the studies used implants containing 30% hydroxyapatite, while the ratios in the other three studies were unknown [31,37,64,85,86]. None of the studies reported extreme reactions.

Another five studies had extensive reactions (45.45%), none of which disclosed the hydroxyapatite ratio [63,71,73,74,80]. Lee et al. (2017) concluded that tibial tunnel widening causes FBR in ACL repairs with PLLA screws, which can be reduced by adding hydroxyapatite. Out of 86 patients in each group, 77 had extensive reactions without hydroxyapatite (89.53%) and 23 had reactions with hydroxyapatite (26.7%).

The one remaining paper reported mild FBR (9.09%). Akagi et al. (2014) found that the closure rate of burr holes from PLLA screws in canine femurs was faster with a 30% hydroxyapatite addition. Additionally, the study reported a decrease in the already mild FBR [18].

Ramsingh et al. (2014) used beta-tricalcium phosphate, produced from hydroxyapatite, as an additive in screws for ACL repair. Out of 268 cases, 14 patients (5.22%) returned with symptoms of swelling, pain, edema, and myxoma [78].

In their 2012 study, Vacanti et al. investigated the effects of adding dexamethasone, a corticosteroid, to subcutaneous fibers in rats. A comparison was made between a group of PLLA-only fibers and three other groups: PLLA fibers with 5.7% dexamethasone, polycaprolactone (PCL) fibers, and PCL fibers with dexamethasone. Although all reactions were mild, the fibers containing PLLA–dexamethasone exhibited the weakest inflammatory response [53]. In a comparable study, Isotalo et al. (1999) compared subcutaneous rods made of PLLA, caprolactone, and caprolactone-coated PLLA in rabbits. The PLLA-only group had the highest, but still mild, FBR at 1 week, but it had the lowest FBR at 1 month [33].

Yang et al. (2020) found that coating PLLA scaffolds with the cytokine macrophage colony-stimulating factor (M-CSF) led to a decrease in pro-inflammatory cytokine levels and an increase in anti-inflammatory cytokine levels in mice [24]. Marascalco et al. (2009) conducted a study on rats with subcutaneous PLLA scaffolds and observed that the intravenous administration of drag reduction polymers (DRPs), such as poly(ethylene oxide) (PEO) and poly(mannose) (PMNN), resulted in a lower FBR and a linearly organized arrangement of newly-formed collagen [47]. Chang et al. (2007) implanted scaffolds containing recombinant bone morphogenetic protein 2 (rhBMP2) into the calves of mice, resulting in the formation of new bones with only mild FBR [46].

### 3.5. Comparisons to Other Biodegradable Materials

Several studies have compared PLLA with other biodegradable materials.

Bergsma et al. (1995) tested different forms of predegraded polylactides, including PLA96, consisting of 96% PLLA and 4% PDLA, CL-PLLA modified by cross-linking, and ordinary PLLA. The study found that PLA96 elicited the strongest immune response, which correlated with faster degradation. In their 1992 study, Majola et al. compared PLLA rods with both poly-D, L-lactic acid (PDLLA) and PLLA rods in rabbit femurs. Neither group exhibited a foreign body reaction. The PLLA group demonstrated superior mechanical strength [29].

In studies comparing reactions to PLLA, polyglycolic acid (PGA), and PGA/PLLA (90:10) pins for ankle repair and various implants, Böstman et al. (1998) and Sinisaari et al. (1995) reported that most foreign body reactions were attributed to PGA [76,81]. Päivärinta et al. (1993) also compared the use of PGA with screws in rat femurs, while Nordström et al. (1998) used pins in rat femurs. Peltoniemi et al. (1999) used screws in sheep skulls, whereas Puumanen et al. (2001) used pins in rabbit backs. All four studies demonstrated a stronger response for the PGA implants [41,44,52,57]. When PLLA, PGA, and polydioxanone (PDS) were compared, Pihlajamäki et al. (2006, 2010) found longer-lasting macrophage activity with PGA and PDS [39,40].

Kang et al. (2007) and Asawa et al. (2012) evaluated poly(lactic-co-glycolic acid) PLGA versus PLLA. They implanted microspheres (50–100 µm) subcutaneously in mice and scaffolds subcutaneously in dogs. In both cases, reactions were more severe with PLGA [19,26].

In their study, Neto et al. (2021) examined the reactions of rats to subcutaneous membranes made from PLLA, biocellulose, and collagen. The results indicated that PLLA membranes were comparable to collagen, while biocellulose membranes exhibited higher levels of FBR [51].

## 4. Discussion

The aim of this systematic review was to classify the risk of a foreign body reaction by a novel anti-infective coating system, which consists of PLLA mixed with adjuvants. Schulze et al. (2022), Pützler et al. (2023), and Schulze et al. (2023) described the coating system [15,16,17], which is filed in a patent application (international publication number: WO2023025944). The functional principle of the coating involves creating a reservoir for the adjuvants through the long degradation time of PLLA. This reservoir is then activated with an extracorporeal shock wave device to release the incorporated adjuvants. During activation, small fragments of PLLA are released as well. The presence of these fragments raised concerns about a potential foreign body reaction.

In a systematic literature review, 71 distinct articles were identified and categorized into four groups according to the severity of FBR. Categorization was based on a review of the symptoms. A reaction was considered absent if there were no symptoms. Long-lasting and damaging clinical symptoms were considered extreme, while other clinical symptoms were considered extensive. Histologic symptoms were classified as mild.

The criteria for determining the presence of a foreign body reaction are not well defined. Some studies have noted the presence of giant cells, but it is not always concluded that they indicate a foreign body reaction. In this categorization, these were considered indicative of a mild reaction. Duranti et al. (1998) established criteria for assessing the severity of an FBR, which includes different grades of severity [88]: (0) no visible reaction; (1) few inflammatory cells; (2) one or two giant cells; (3) fibrous tissue with inflammatory cells, lymphocytes, and giant cells; and (4) granuloma with capsule. The present categorization, which considers the possible clinical complications, considers all these grades as mild. However, from a histological perspective, a multilevel classification of the severity of FBR is useful and should be combined with the grades of clinical complications in clinical practice.

Regarding absent reactions, 28.17% of the articles were classified as such. Additionally, 7.04% and 11.27% were classified as extreme and extensive, respectively. The majority of articles (54.93%) were found to have mild reactions.

Seven out of twenty articles in the group without FBRs did not conduct histology. According to the criteria used here, it is possible that a mild FBR was present, which would add another 9.86% to this category. In Illi et al. (1994), histology was performed only after the implant had been completely absorbed [83]. Since FBR ceases once the PLLA is degraded, mild FBR may have existed before. Other articles primarily focused on the bone, and it is unclear how closely the soft tissue histology was examined. If it was ignored, the possibility of a mild reaction also applies here.

Within the group of extreme reactions, three distinct symptoms were observed: sinus formation, osteolysis, and Oppenheimer effect sarcomas. The occurrence of osteolysis in three of the articles can be explained by the degradation process of PLLA. The degradation of PLLA results in the accumulation of lactic acid, which is subsequently broken down in the body by the liver or mitochondria-rich tissues into carbon dioxide and water [4,5]. Due to the lower blood supply and fewer mitochondria in bones than in soft tissues, the breakdown of lactic acid takes longer in bones. Local acidosis triggers osteoclastic bone resorption in alkaline bones [89]. To prevent this, the literature suggests adjusting the crystallinity or adding alkaline particles like hydroxyapatite to the PLLA [90,91].

The Oppenheimer effect refers to the development of sarcoma in rodents due to the presence of a foreign body. Oppenheimer et al. (1958) and Ott (1970) found that the occurrence of these tumors is not dependent on the chemistry of the foreign body. However, the size, form, and duration are all important factors. A faster degradation and a higher inflammatory response may inhibit the formation of sarcomas [55,92,93]. Due to their limited occurrence in specific species, they are not a concern in the clinical application of the coating system. However, it is important to consider this effect in pre-clinical in vivo models.

Extensive symptoms included redness, tenderness, swelling, bone marrow edema, ganglion cysts, and nodules. Five of the eight articles examined fewer than 30 subjects, and some had high rates of extensive reactions. Six of the articles examined fewer than 30 subjects, which may have compromised the validity of the studies due to the small sample size. Facial plates were associated with redness, tenderness, and swelling, while screws used during ACL repair were associated with bone marrow edema and ganglion cysts. Nodules were only observed in one study that used filler.

In vivo studies showed only mild or absent foreign body reactions, except for the sarcomas caused by the Oppenheimer effect. This raises the question of whether in vivo models are effective in predicting foreign body reactions in clinical settings. It is possible that in vivo trials are too controlled, while clinical situations are more unpredictable. However, it is also possible that the number of animals used in the trials may be insufficient for accurate predictions. Only five studies tested more than 50 animals.

### 4.1. Location as a Factor

When considering localization as a factor, it appears that juxta-articular regions and other areas with high mobility, such as the face, may cause issues. One possible explanation is that the strong friction on the polymer leads to faster degradation and a stronger reaction. Moreover, the tissue may already be irritated by the friction. Additionally, insertion into the bone seems to be disadvantageous due to tunnel widening, which can lead to strong reactions when screws are inserted, as described by Lee et al. (2017) [74]. As previously discussed, the pH difference between lactate and bone has been identified as a common cause of osteolysis [89,90]. However, the developed coating system is currently only intended for application to soft tissue-adjacent surfaces. The activation of the coating through extracorporeal shock waves is only effective when propagating through soft tissue and not through bone. Therefore, this should not pose a problem.

### 4.2. Implant and Particle Parameters as a Factor

Bergsma et al. (1995) reported a particle size of 2.742 mm^2^ after degradation [45]. Zhang et al. (2021) also provided information on the size of individual microspheres in their produced fillers, which ranged from 5 to 100 µm [7]. Both articles were classified as mild reactions. According to Morhenn et al. (2002), microspheres with a diameter greater than 40.2 mm cannot be phagocytized, and the likelihood of larger particles inducing a reaction is low [94]. Isotalo et al. (1999) reported that phagocytosis began with particles ranging from 10 to 80 µm, while Baranov et al. (2020) reported that phagocytosis was most efficient with particles of approximately 3 µm [33,95]. 

The particles produced by the activation of the novel PLLA coating range from 0.5 to 4 µm, which should result in very efficient phagocytosis. Successful phagocytosis by macrophages may prevent the emergence of foreign body giant cells. Consequently, the reaction should be mild according to the proposed classification and grade 1 of the criteria suggested by Duranti et al. (1998) [88].

Contradictions exist regarding the relationship between the reaction and molecular weight. Pistner et al. (1998) reported that the mildest FBR was associated with the lowest molecular weight, while Zhang et al. (2021) found that the mildest FBR and slowest degradation were associated with the highest molecular weight [7,55]. It is commonly believed that the degradation of PLLA accelerates as the molecular weight increases [9]. The difference in degradation observed in the latter article may be due to the form of the implanted PLLA, a filler. This could be due to the fact that the filler is composed of microspheres of varying sizes and is not a solid implant. In this explanation, the degradation of microspheres is influenced more by their size than their molecular weight.

If this is generally true, a lower molecular weight would be beneficial in producing a less pronounced reaction. Due to the proportionality to intrinsic viscosity and inverse proportionality to the degree of crystallinity, a lower viscosity and higher degree of crystallinity would be equally beneficial. However, it is commonly believed that higher crystallinity is responsible for stronger foreign body reactions, as described by Kim et al. (2019) [73]. Considering the different behavior of the filler in Zhang et al. (2021), it is more likely that a higher molecular weight, higher viscosity, and lower degree of crystallinity are beneficial [7].

Overall, the papers suggest that a slower degradation process, resulting in lower molecular weight and viscosity, leads to a lower severity of the FBR. It appears that higher crystallinity may be beneficial, although this is not widely agreed upon [73]. To explain the degradation, it should be analyzed in two parts. The initial stage involves the gradual degradation of the amorphous components of the polymer. This process should occur at a slow rate to avoid any adverse effects on the surrounding tissue. The polymer’s crystalline components remain. As per Suuronen et al. (1998), these crystalline fragments are not soluble in water and, therefore, should be phagocytosed [60]. In order to avoid the formation of giant cells, it is necessary to have specific fragment sizes. Therefore, to optimize the degradation curve of PLLA, it is suggested to find a balance between the molecular weight and the degree of crystallinity. This balance can be achieved by using different methods of processing the polymer. For instance, achieving the same resulting molecular weight by injection molding and electrospinning would not have the same degree of crystallinity. Additionally, the resulting structure can be beneficial for biocompatibility [96]. To classify this in the novel anti-infective coating, it is imperative to determine the values of the PLLA used.

### 4.3. Duration of Implantation as a Factor

There is no conclusive evidence regarding the implantation time that leads to a severe foreign body reaction. However, some studies have shown a decreasing effect after varying implantation times, which is likely due to differences in degradation rates. In the literature, the waning effect of a typical foreign body reaction has been described as its second phase [4,5,10,11]. It is commonly reported that the reaction subsides after degradation is complete [13]. This is supported by the reviewed studies. In cases of extreme or extensive reactions, the removal of the implant was found to be the most effective treatment in all cases.

### 4.4. Additives as a Factor

Several studies investigated additives to the PLLA, most commonly hydroxyapatite. Hydroxyapatite is a mineral that has been widely used as an additive in PLLA implants for the past two decades. It promotes osteoconductivity because it is very similar to bone mineral. Additionally, it reduces the hydrophobicity of PLLA, allowing for quicker hydrolysis. The implant also possesses a strength similar to that of bone with added HA [91,97]. Akagi et al. (2014) demonstrated the usefulness of hydroxyapatite in promoting faster burr hole closure and lower FBR [18]. Hydroxyapatite is particularly beneficial for implants placed directly into the bone, such as screws or anchors. However, in soft tissue, it may accelerate implant degradation.

### 4.5. Comparisons to Other Biodegradable Materials

When comparing the reactions of PLLA and other biodegradable polymers, it appears that the purity of PLLA is a crucial factor. When compared with PLLA, Bergsma et al. (1995) found a stronger reaction of PLA96, a mixed form of the L- and D-enantiomers of PLLA [45]. The rate of degradation of this polymer is considerably faster than that of PLLA. Therefore, the stronger foreign body response can probably be attributed to faster degradation. Additionally, Kang et al. (2007) and Asawa et al. (2012) observed that PLGA, which is a polymer of lactic acid and glycolic acid, also elicited a stronger response [19,26]. Other studies have also reported stronger reactions to PGA, which likewise has a higher degradation rate than PLLA, resulting in a stronger response [39,40,41,44,52,57,76,81].

### 4.6. Summary of the Factors

To summarize, this implant coating system does not pose any issues with locations that are problematic when in contact with PLLA, such as juxta-articular regions, the face, and general bone contact, as they are not intended to be coated. The size of the fragments created by the coating’s activation (ranging from 0.5 to 4 µm) closely matches the size of the most efficient phagocytosis (3 µm) and should thus not elicit a reaction. To prevent a strong reaction, degradation should occur slowly through the use of low molecular weight and low viscosity. This degradation should be compatible with the intended release of the drug by the degradation process. To prevent the formation of highly crystalline fragments, there should be a balance between the degree of crystallinity and the molecular weight, which are inversely proportional. The duration of implantation does not appear to correlate with the severity of the reaction. However, in rare cases, the removal of the PLLA might be indicated, leading to restitution. Hydroxyapatite is a useful additive for bone-related usage but not for soft tissue. Ultimately, the PLLA should be as pure as possible to ensure slow degradation.

## 5. Conclusions

In this systematic review, mild reactions were the most common occurrences. Major complications were rare and could be resolved by removing the implanted material. The type of biomaterial in contact with the PLLA implant seems to be responsible for stronger reactions. If used as a filler for plastic purposes, mild reactions may even be desirable to induce collagen production. In bone, local acidosis accelerates degradation, which is associated with osteolysis. Hydroxyapatite is one approach to improving the biocompatibility of PLLA in bone. In addition, locations with high mobility and thus friction and faster degradation, such as joints and the face, may be unsuitable.

The current review supports estimating the new coating’s potential risk for foreign body reaction in its intended use for contact with soft tissue. It is important to note that, at this point, the implants’ articulation and bone contact areas are not intended to be coated. Furthermore, the fragments of PLLA that result from activation are approximately 0.5 to 4 µm in diameter, which should allow for unproblematic phagocytosis and thus a very mild reaction. For further classification, the molecular weight, intrinsic viscosity, and degree of crystallinity of the PLLA used should be determined.

## 6. Patents

A patent application has been filed for the coating (international publication number: WO2023025944).

## Figures and Tables

**Table 1:** List of articles with absent foreign body reactions (28.17%).

First Author	Year	Study Type	Organism	Location	Implant	Additive	n	
Majola A.	1992	in vivo	rabbit	femur	rod	n/a	40	[29]
Yamamuro T.	1994	clinical (retrospective)	human	various	various	n/a	143	[72]
Illi O.E.	1994	clinical	human	various	plug/nut	n/a	32	[83]
Kunz E.	1995	in vivo	rabbit	subcutaneous leg /muscle/tibia	rod	n/a	20	[43]
Koskikare K.	1996	in vivo	rabbit	femur	plate	n/a	20	[34]
Burns A.E.	1998	clinical (prospective)	human	metatarsus	rod	n/a	25	[66]
Pelto-Vasenius K.	1998	clinical (retrospective)	human	ankle	pin/screw	n/a	30	[77]
Weir N.A.	2004	in vivo	rat	subcutaneous back	rod	n/a	12	[54]
Handolin L.	2005	clinical (prospective)	human	ankle	screw	n/a	22	[67]
Handolin L.	2007	in vivo	rat	femur	rod	n/a	32	[49]
Pihlajamäki H.K.	2010	in vivo	rabbit	femur	pin	n/a	80	[40]
Stein T.	2011	clinical (prospective)	human	shoulder joint	anchor	n/a	37	[68]
Saadai P.	2011	in vivo	sheep	spine	scaffold	n/a	2	[62]
Stein T.	2012	clinical (prospective)	human	shoulder joint	anchor	n/a	53	[65]
Morizane K.	2019	in vivo	rabbit	knee	rod	hydroxyapatite	32	[37]
Sukegawa S.	2019	clinical	human	facial	screw	hydroxyapatite	5	[85]
Morizane K.	2021	in vivo	rabbit	femur/tibia	pin	hydroxyapatite	32	[31]
Usami T.	2021	clinical	human	patella	screw	hydroxyapatite	15	[86]
Oishi K.	2023	clinical (prospective)	human	sternum	plate	hydroxyapatite	39	[64]
Zhou S.-Y.	2023	in vivo	mouse	subcutaneous back	filler	n/a	8	[27]

**Table 2:** List of articles with extreme foreign body reactions (7.04%).

First Author	Year	Study Type	Organism	Location	Implant	Additive	n	Symptoms	
Pistner H.	1993	in vivo	rat	subcutaneous back/muscle	rod/cube	n/a	67	immune cells, FBGCs, fibrous capsule; 3 Oppenheimer effect sarcomas	[55]
Sinisaari I.	1995	clinical	human	various	various	n/a	420	1 patient with sinus formation	[81]
Tams J.	1996	clinical	human	facial	plate	n/a	4	1 patient with immune cells, FBGCs, swelling, osteolysis	[82]
Böstman O.M.	1998	clinical (retrospective)	human	ankle	pin/screw	n/a	234	1 patient with ankle arthrodesis; osteolysis, cystic extensions, FBGCs	[76]
Potapov A.	2011	clinical (retrospective)	human	biceps tendon	screw	n/a	22	1 patient with sinus, osteolysis, swelling, tenderness	[75]

**Table 3:** List of articles with extensive foreign body reaction (11.27%).

First Author	Year	Study Type	Organism	Location	Implant	Additive	n	Symptoms	
Bergsma E.J.	1993	clinical	human	facial	plate, screw	n/a	10	9 patients with swelling, immune cells, FBGCs, fibrous capsule	[79]
Hayashi M.	2013	clinical	human	facial	plate, screw	hydroxyapatite	17	2 patients with inflammatory granulation tissue, 1 patient with swelling also	[80]
Landes C.	2014	clinical (prospective)	human	facial	plate	hydroxyapatite	29	2 patients with swelling	[63]
Janjua T.A.	2014	clinical (prospective)	human	various	filler	n/a	91	27 patients with massage nodules; 8 patients without massage nodules	[70]
Ramsingh V.	2014	clinical (retrospective)	human	ACL	screw	tri-calcium phosphate	268	14 patients with swelling, pain, edema, myxoma (cysts)	[78]
Lee D.W.	2017	clinical (retrospective)	human	ACL	screw	n/a/hydroxyapatite	86/86	66 edema + 11 cysts in PLLA/21 edema + 2 cysts in PLLA-HA	[74]
Kim Y.M.	2019	clinical (retrospective)	human	facial	plate	hydroxyapatite	15	3 patients with redness, tenderness	[73]
Veizi E.	2022	clinical (retrospective)	human	ACL	screw	hydroxyapatite	27	9 patients with edema + 2 patients with tibial tunnel cysts	[71]

**Table 4:** List of articles with mild foreign body reaction (54.93%).

First Author	Year	Study Type	Organism	Location	Implant	Additive	n	Symptoms	
Schakenraad J.M.	1988	in vivo	rat	subcutaneous back	fiber	n/a	78	immune cells, fibrous capsule	[48]
Bos R.R.M.	1991	in vivo	rat	subcutaneous back	plate	n/a	35	immune cells, fibrous capsule	[6]
Matsusue Y.	1992	in vivo	rabbit	subcutaneous back/knee	rod	n/a	70	fibrous tissue	[30]
Manninen M.J.	1992	in vivo	rabbit	femur	rod	n/a	42	immune cells, FBGCs	[36]
Kinoshita Y.	1993	in vivo	dog	subcutaneous back	mesh	n/a	22	immune cells, FBGCs, fibrous capsule	[20]
Laitinen O.	1993	in vivo	sheep	ACL	fiber	n/a	32	immune cells, FBGCs, fibrous tissue	[61]
Manninen M.J.	1993	in vivo	rabbit	tibia	screw	n/a	36	immune cells, FBGCs	[35]
Päivärinta U.	1993	in vivo	rabbit	femur	screw	n/a	25	immune cells, FBGCs	[44]
Bergsma J.E.	1995	in vivo	rat	subcutaneous back	capsule	n/a	20	immune cells, FBGCs, fibrous capsule	[45]
Peltoniemi H.H.	1998	in vivo	sheep	cranium	plate	n/a	9	immune cells, FBGCs, fibrous capsule	[58]
Peltoniemi H.H.	1998	in vivo	sheep	cranium	plate	n/a	6	immune cells, FBGCs, fibrous capsule	[59]
Suuronen R.	1998	in vivo	sheep	facial	plate	n/a	5	immune cells, FBGCs	[60]
Nordström P.	1998	in vivo	rat	femur	pin	n/a	51	immune cells, FBGCs, fibrous tissue	[52]
Korpela A.	1998	in vivo	rabbit	trachea	stent	n/a	9	immune cells	[42]
Peltoniemi H.H.	1999	in vivo	sheep	cranium	screw	n/a	20	immune cells, FBGCs, fibrous capsule	[57]
Isotalo T.	1999	in vivo	rabbit	subcutaneous back	rod	n/a/caprolactone	15	immune cells, FBGCs, fibrous tissue	[33]
Peltoniemi H.H.	1999	in vivo	sheep	cranium	screw	n/a	10	immune cells, FBGCs, fibrous capsule	[56]
Hooper K.A.	2000	in vivo	rat	subcutaneous back	disc	n/a	n/a	immune cells, FBGCs	[50]
Puumanen K.A.	2001	in vivo	rabbit	back with tibial graft	pin	n/a	12	immune cells	[41]
Parker J.A.T.C.	2002	in vivo	goat	subcutaneous flank	disc	n/a	6	immune cells	[22]
Parker J.A.	2002	in vivo	goat	subcutaneous flank/subperiosteal frontal bone	anchor	n/a	18	immune cells, FBGCs, fibrous capsule	[23]
van Dijk M.	2005	in vivo	goat	vertebra	cage	n/a	43	immune cells, FBGCs	[21]
Pihlajamäki H.	2006	in vivo	rabbit	femur	screw	n/a	32	immune cells	[38]
Pihlajamäki H.	2006	in vivo	rabbit	knee	pin	n/a	20	immune cells, fibrous tissue	[39]
Shumaker P.R.	2006	in vivo	pig	abdomen	filler	n/a	1	immune cells, FBGCs	[28]
Chang P.C.	2007	in vivo	rat	calf	scaffold	rhBMP2	20	immune cells, FBGCs	[46]
Kang S.-W.	2007	in vivo	mouse	subcutaneous back	microsphere	n/a	28	immune cells	[26]
Marascalco P.J.	2009	in vivo	rat	subcutaneous back	scaffold	n/a	15	FBGCs, new collagen	[47]
Fujihara Y.	2010	in vivo	mouse	subcutaneous back	scaffold	n/a	3	immune cells, FBGCs, fibrous tissue	[25]
Asawa Y.	2012	in vivo	dog	subcutaneous	scaffold	n/a	6	immune cells, cytokines	[19]
Sena P.	2012	clinical	human	foot	screw	n/a	33	13 patients with immune cells, FBGCs	[84]
Vacanti N.M.	2012	in vivo	rat	subcutaneous back	fiber	n/a/dexamethasone	36	immune cells, fibrous capsule	[53]
Akagi H.	2014	in vivo	dog	femur	screw	hydroxyapatite	12	immune cells, fibrous tissue	[18]
Werner M.	2014	clinical (prospective)	human	artery	stent	n/a	30	4 patients with immune cells, 2 with FBGCs also	[69]
Yang N.	2020	in vivo	mouse	subcutaneous back	scaffold	PIII + M-CSF	20	FBGCs, fibrous capsule, cytokines	[24]
Zhang Y.	2021	in vivo	rabbit	subcutaneous back	filler	n/a	21	immune cells, FBGCs, fibrous capsule	[7]
Neto J.D.	2021	in vivo	rat	subcutaneous back	membrane	n/a	15	immune cells, fibrous tissue, granulation tissue	[51]
Sun L.	2023	in vivo	rabbit	subcutaneous back	filler	n/a	12	immune cells, FBGCs, fibrous tissue	[32]

## Data Availability

The original contributions presented in the study are included in the article; further inquiries can be directed to the corresponding author.
